# Content-Based Audio Classification and Retrieving Using Modified Bacterial Foraging Optimization Algorithm

**DOI:** 10.1155/2023/7735846

**Published:** 2023-07-07

**Authors:** Amani K. Samha, Ghalib H. Alshammri, Stephen Jeswinde Nuagah, Madhu Sharma, R. Mekala

**Affiliations:** ^1^Management Information System Department, College of Business Administration, King Saud University, Riyadh 28095, Saudi Arabia; ^2^Department of Computer Science, Community College, King Saud University, Riyadh 11437, Saudi Arabia; ^3^Department of Electrical Engineering, Tamale Technical University, Tamale, Ghana; ^4^School of Engineering, University of Petroleum and Energy Studies, Dehradun, Uttarakhand 248001, India; ^5^Department of Information Technology, M.Kumarasamy College of Engineering, Thalavapalayam, Karur, Tamil Nadu, India

## Abstract

Audio classification and retrieval has been recognized as a fascinating field of endeavor for as long as it has existed due to the topic of identifying and choosing the most useful audio attributes. The categorization of audio files is significant not only in the area of multimedia applications but also in the disciplines of medicine, sound analysis, intelligent homes and cities, urban informatics, entertainment, and surveillance. This study introduces a new algorithm called the modified bacterial foraging optimization algorithm (MBFOA), which is based on a method that retrieves and classifies audio data. The goal of this algorithm is to reduce the computational complexity of existing techniques. Along with the combination of the peak estimated signal, the enhanced mel-frequency cepstral coefficient (EMFCC) and the enhanced power normalized cepstral coefficients (EPNCC) are used. These are then optimized using the fitness function utilizing MBFOA. The probabilistic neural network is used to differentiate between a music signal and a spoken signal from an audio source (PNN). It is next necessary to extract and list the characteristics that correspond to the class that was arrived at as a consequence of the categorization. When compared to other approaches that are somewhat similar, MBFOA demonstrates superior levels of sensitivity, specificity, and accuracy.

## 1. Introduction

Retrieving audio information automatically has gained a lot of reputation for organizing a wide variety of audio files in the database. However, the current process of audio retrieval does not characterize the perceptual similarity of the audio signals, as they depend primarily on the measure of similarity. This article proposes an approach to audio classification and retrieval based on MBFOA to overcome the above issues. Using MBFOA along with the fitness function calculation, selecting the optimum feature is an easy job. To classify the audio signal as a music or speech signal, PNN is performed. The audio signal category is then extracted from the result of the classification. Then the calculation of the Euclidean distance is performed, and the relevance matching is done to retrieve the files related to the query. Finally, it is ensured that the proposed approach is consistent with the existing approach.

Audio signals, such as voice, music, and the sounds of the environment, are essential examples of many sorts of media. The difficulty of separating audio signals into the many forms of audio being produced is thus becoming an issue of growing significance. Simply listening to a brief portion of an audio signal is sufficient for a human listener to be able to correctly identify a wide variety of audio formats. However, finding a solution to this issue via the use of computers has proved to be quite challenging. Despite this, a large number of systems with just a moderate degree of precision might still be constructed. The processes of audio segmentation and classification have a broad range of potential applications. For example, content-based audio categorization and retrieval is utilized extensively in the entertainment sector as well as in the administration of audio archives and the use of commercial music, surveillance, and other fields. Nowadays, numerous digital audio databases can be found on the World Wide Web; in these cases, audio segmentation and classification are required in order to do audio searching and indexing. There has been a significant uptick in interest in monitoring broadcast news programmes as of late. In this context, the categorization of speech data in terms of speaker might be of assistance in facilitating the effective navigation of broadcast news archives.

A signal processing part and a classification section are the two primary components of any audio classification work, just as they are in the majority of other pattern classification endeavors. The extraction of characteristics from the audio stream is the focus of the signal processing portion of the algorithm. In many situations, the different techniques of time-frequency analysis that were first established for speech processing are applied. These approaches were designed for the purpose of processing audio data. The classification stage involves assigning categories to collected data based on the statistical information that was derived from the signals.

The amount of digital music files stored on personal computers has expanded as a direct result of the expansion of the market for portable digital audio players. When you want to listen to music that fits a certain mood, such as happy music, sad music, or furious music, it may be challenging to pick and organize the songs that you want to listen to in order to achieve that mood. Consumers are not the only ones who are required to label their music; online distributors also have to label the thousands of tracks that are stored in their databases for their customers to peruse.

In the fields of music psychology and music education, emotion-based musical components have been identified as the aspect of music that is most closely related to the concept of music expressivity. Music information behaviour research has also highlighted music mood emotion as an essential factor that individuals employ while searching for music and storing it. This criterion is utilized by music indexing and storage. However, because of the great degree of subjectivity involved, determining the atmosphere of a piece of music may be challenging. It is possible to infer aspects of a person's personality based on the music to which they listen. While we listen to music, there are a number of different elements that subtly but significantly alter it. Some examples of such things are rhythm, pace, musical instruments, and musical scales. Lyrics are a very significant entity that has a direct impact on our thoughts, and they may be found in a variety of forms.

The task of extracting audible words from lyrics and then categorizing the music in accordance with those words is a challenging one since it involves intricate problems with digital signal processing. A rather straightforward method of comprehending the music based on its acoustic patterns was investigated using these processing techniques. This difficulty is similar to the traditional one of pattern recognition, and people have been working hard to isolate these patterns from the audio elements.

Through the use of an artificial neural networking system, a computer can, in all seriousness, read the thoughts of humans. In spite of the fact that a computer is responsible for the dot's gyrations, the machine was only obeying the directions given to it by the subject of the experiment. The method of artificial neural networks is much more than just a trick performed in the lab. Despite the fact that computers are capable of solving issues that are very complicated quickly, standardized assessments of one's conduct may be found in the field of psychology. The vast majority of psychological tests may be broken down into two primary groups: mental ability measures and personality scales. Intelligence tests, which are intended to assess an individual's overall mental ability, and aptitude tests, which examine an individual's specialized mental talents, are both included in the battery of mental ability tests. Since there is no one correct or incorrect response to a personality scale, this kind of assessment is more often referred to as a scale. Various reasons, interests, values, and attitudes may all be measured via the use of personality tests. The findings of the experiment demonstrate that the ANN-MFCC method system is successful.

The authentication process that the user goes through is often either unusually lax or very stringent, depending on the situation. Authentication has been an intriguing method all through the years that it has been used. Because of the proliferation of technological tools, it is becoming simpler for “others” to create false identities, steal identities from others, or crack the passwords of others. As a consequence of this, many algorithms have been developed, each of which takes an intriguing approach to the computation of a secret key. The algorithms are designed in such a way that they select a random number somewhere between 10 and 6.

Users in the modern day are given main password stereotypes such as textual passwords, biometric scanning, tokens or cards (such as those used at an ATM), and other similar options. The majority of textual passwords are encrypted using the same method that was discussed before. Your “natural” signature is obtained by biometric scanning, and cards or tokens are used to verify your identity. But some individuals despise having to carry their cards with them and others refuse to put their retinas through intense IR radiation (biometric scanning). The majority of textual passwords used these days are kept very basic, such as a word from the dictionary, the name of a pet, or the name of a lover. Several years ago, Klein carried out these experiments, and he was able to break 10–15 passwords in a single day. This is now quite easy to do because of improvements in technology such as faster processors and an abundance of resources available on the Internet.

As a result, with our current notion, this system protects the 3D passwords, which are a particularly intriguing method of authentication because of their increased level of personalization. The number of possible passwords has been increased to 21, considering the limits of human memory. In most cases, basic passwords are used so that the information may be easily recalled. In our system, the human memory is required to go through the processes of recognition and recall, as well as any biometric or token-based authentication. After it has been installed and you have logged in to a secure website, the graphical user interface for the 3D password will appear. This is an extra password that the user may just type in. It is a textual password. After he passes the first verification, a 3D virtual area will become accessible to him on the screen. In our scenario, let us assume a virtual garage. Now, in a typical garage, one can discover a wide variety of tools, pieces of equipment, and other items, all of which have their own specific qualities. Thereafter, the user will interact with these attributes appropriately. Each item in the three-dimensional space may be moved freely in any of the three planes *x*, *y*, and *z*. That is the property of each thing that allows it to move. This quality is shared by all of the items that are located in the space. Let us say a person successfully logs on and navigates their way into the garage. He notices and picks up a screwdriver, which is the beginning position in *xyz* coordinates (5, 5, 5), and then he moves the screwdriver five spaces to his right (on the *xy* plane, this would be 1) (10, 5, 5). That is a kind of authentication that can be recognized. The actual user is the only one who can comprehend and identify the thing that he must choose from among numerous others. This is when the “recall and recognition” section of a person's memory comes into action. It i's interesting to note that a password may be established by walking up to a radio and changing the frequency to a number that the user alone is aware of. The fact that cards and biometric scanners may be used as input can help to increase the system's level of security. A user may be subjected to a variety of different degrees of authentication.

## 2. Related Works

In this part of the article, the various strategies and procedures for the regular research effort linked with the categorization and retrieval of audio signals are discussed. The categorization and segmentation of audio was accomplished via the use of ensemble techniques [1]. The signals may be broken down into speech, music, sounds of the surroundings, speech that has been isolated from other sounds, and silence. The zero-crossing rate (ZCR), the short-time energy (STE), the spectrum flux, the cepstral coefficients of mel-frequency (MFCC), and the analysis of periodicity were all used in order to extract features. When attempting to categorize the music and surrounding noises, it is possible to produce discernible inaccuracies.

Pitch density-based parameters are used in [2], such as the average pitch density and relative tonal density. With the use of the pitch density-based features, a multi-class coarse-to-fine classification that was both highly accurate and more efficient than before was accomplished. The development of a comprehensive and one-of-a-kind approach for extracting audio characteristics was undertaken to achieve a high classification accuracy for ambient audio data. The classification accuracy of the suggested technique has been increased when compared to the MFCC features [3]. The Gammatone cepstral coefficients, also known as GTCCs, were used in order to categorize the audio signals that did not originate from speech. It was discovered that the classification accuracy was much greater when compared to other conventional audio characteristics. Particularly at low frequencies [4], it was discovered that the GTCC was much more effective than the MFCC.

As part of the content-based retrieval (CBR) work that the Muscle fish Company was doing, they repeated the process of measuring the statistical values in the frequency domain multiple times in order to quantify perceptual characteristics including loudness, brightness, bandwidth, and pitch. This method is only useful for analyzing timbre sounds [5] due to the ease with which the approach of employing simply statistical data can be carried out. In content-based audio retrieval, the query-by-humming approach is one of the particular techniques employed. The strategy [6] employed string-matching techniques for detecting which songs are similar to one another and described the sequence of relative pitch fluctuations that would be used to depict the contour of the melody. A transition in pitch between 10 and 12 has been noted for it.

A one-by-one comparison was made between the displayed signal and each signal in the database. The sample from the database is retrieved [7] if the same requirements are satisfied as before. The following is an explanation of the retrieval system's fundamental workings. First, an estimate is made [8] of the feature for each user signal as well as the signal from the database. When it comes to audio signals, the temporal domain is the same as their original domain. One thing that all temporal choices have in common is that they are taken from the received raw audio data in its original form without undergoing any transformations beforehand.

The organizations can better filter out anonymous calls with the use of voice and audio recognition done in real time. For content-based music information retrieval, a k-nearest neighbor learning technique based on a support vector machine has been presented [9]. When there are a significant number of audio files of varying sizes, the use of approaches that utilize machine learning is recommended [10]. In order to manage such large amounts of audio data, a decision tree classifier based on AdaBoost has been constructed [11]. Recognizing and classifying sounds is an extremely important step in the process of developing assistive technology for persons with hearing impairments. When classifying audio events, convolutional neural networks (CNN) combined with pretrained VGG networks are employed to extract feature embeddings [12].

The identification of bird songs may also be accomplished with the help of multi-modal deep CNN by using audio samples and metadata as inputs. Utilizing quadratic time-frequency distributions [13], an audio surveillance system has been developed to prevent automobile collisions and tyre sliding in hazardous situations. The effective music indexing framework (EMIF) has been created [14] with the goals of increasing the efficiency of queries and facilitating the retrieval of scalable and accurate music. It does this by applying semantic-sensitive categorization to the music in order to determine its categories. The content-based music retrieval tool known as Pandora radio takes use of the commonalities between song characteristics including genre, mood, instrumentation, and voice quality [15]. In contrast to text files, audio recordings are stored in an unstructured manner, which necessitates the use of more complicated algorithms during preprocessing and categorization [16]. In this instance, psycholinguistic and stylistic characteristics were used in order to categorize auditory emotion. The following is the contribution of this article:

- A whole new audio file format is suggested by this work
- A new method for the categorization of audio files and the retrieval of their contents is proposed in this study.
- The proposed method attains excellent levels of sensitivity, specificity, and accuracy in comparison to the current method.
- Experiments are carried out using a dataset that is considered standard.
- The computational complexity of methods for classifying data that make use of probabilistic neural networks is simplified thanks to this strategy.
- This scheme may be used to identify the audio signals in a wide variety of applications including multimedia.
- Individuals who struggle with their hearing may find a significant improvement in their quality of life after implementing this plan.

## 3. Materials and Methods

**Figure 1:**
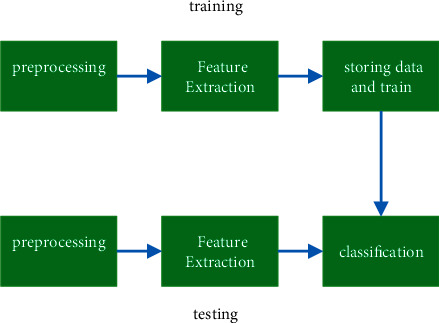
Flow diagram of the audio classification and retrieval.

This section provides a detailed explanation of the classification and retrieval strategy that will be used for the anticipated audio stream. A mean filter is used in order to accomplish the smoothing of audio data. In order to extract the retrieval stage feature, EMFCC-EPNCC [17] is used, in conjunction with a mixture of peak estimated signal characteristics. Using the MBFOA method, the feature vector that was produced is analyzed so that the best possible feature index may be chosen [18]. The suggested method of categorizing audio and retrieving it is shown in the flow diagram in Figure 1, which may be found here.

### 3.1. Segmentation and Feature Extraction

During this stage of peak feature extraction, the notation *R* Loc refers to the features that are located within the high peak of the positive edge of the audio signal, *Q* Loc refers to the features that are located at the minimum signal difference within the negative edge of the audio signal, and *S* Loc represents the features that are located at the maximum signal difference within the negative edge of the signal [19]. In order to update the difference in peak extraction, the threshold value that was retrieved from the input signal is applied. The study that was suggested [20] includes references to the processes of pitch extraction and peak extraction. The objective function is first used during the pitch extraction process to determine the weight of the signal based on the difference in the cosine angle of the amplitude of the signal [21]. This is done by applying the objective function at the beginning of the procedure. The pitch of the audio stream may be approximated by using a detection approach that is based on the time domain.

Many different approaches are used to estimate pitch. Some of these methods are the zero-crossing method, the autocorrelation method, the maximum likelihood method, the adaptive filtering method employing fast Fourier transform (FFT), and the super-resolution pitch detection method [22]. The audio signal characteristics are extracted with the help of EMFCC-EPNCC. These features make up a set that is mostly used to represent the audio signal. When determining the feature values to return, the extraction of features considers EMFCC and EPNCC. These feature values are derived from the audio signal and sampled at *fs* (Hz). The windowing approach is used to the audio stream in order to break it up into individual frames and do spectrum analysis on each frame. The frame was then applied to the discrete Fourier transform at that moment in time (DFT). Thereafter, the output of the DFT is distorted due to warping [23].

The window distribution is characterized as

(1) $$\begin{array}{c} P_{i}\left(k\right)=\left(\frac{1}{N}\right){\left|X_{i}\left(k\right)\right|}^{2}\;. \end{array}$$

The estimate of the power spectrum is denoted by the variable *P*_*i*_(*k*), which may take the values *k*=0,  1,   …,  [(*N*/2) − 1]. *N* is the total number of samples that were taken. The change in pitch angle is determined for each pre-allocated time sample by using the size of the input signal *X*_*i*_ as the starting point [24].

The definition of the function of the frequency warping intensity of the *mel* may be found in

(2) $$\begin{array}{c} t^{\prime }\left(\omega \right)=2595\cdot \;\log\left(1+\frac{\omega f_{s}}{2\pi \cdot 700Hz}\right). \end{array}$$

In this context, the sampling frequency is denoted by *fs*, and the function of frequency received is denoted by *t*. In order to fulfil the particular requirements, it is necessary to standardize the function of *mel*-warping. *p*(*π*) = *π*.

(3) $$\begin{array}{c} t^{\prime }\left(\omega \right)=\frac{\pi }{\mu \left(\pi \right)}\times \mu \left(\omega \right)d\cdot \;\log\log\left(1+\frac{\omega f_{s}}{2\pi \times 700Hz}\right), \end{array}$$

where

(4) $$\begin{array}{c} d=\frac{\pi }{\log\left(1+f_{s}/2\times 700Hz\right)}. \end{array}$$

### 3.2. Feature Selection and Classification

After employing the MBFOA selection of features to pull a collection of testing features out of the audio file, the optimal testing features are then chosen for selection. The colony behaviours of *Escherichia coli* (*E. coli*) demonstrate that these bacteria are highly organized and capable of forming complex structures [25]. Passino [15] developed a brand-new technique for optimization that he called the bacterial foraging (BF) algorithm [26]. This algorithm was inspired by the foraging behaviour of the *E. coli* bacterium. Four primary phases are used in the process of determining the foraging behaviour of bacteria, which include chemotaxis, swarming, reproduction, and elimination and dispersion.

An MBFOA [27] that has an improved fitness function computation is employed in the process of selecting the best possible value for each feature [28]. MBFOA required fewer user parameters to be specified while producing results that were comparable to those of other methods, and it reduced the total number of function evaluations performed. The elimination of user-defined parameters and the presence of an internal BFOA loop are the primary benefits of MBFOA [29].

The bacterial set is initialized by using:

(5) $$\begin{array}{c} G\left(x\right)=\left\{g_{1}\left(x\right),g_{2}\left(x\right),\ldots ,g_{n}\left(x\right)\right\}. \end{array}$$

The pareto optimal set is found as follows:

(6) $$\begin{array}{c} G_{\min}\left(x\right)={\left\{g_{1}\left(x\right),g_{2}\left(x\right),\ldots ,g_{n}\left(x\right)\right\}}^{T}, \end{array}$$

where *g*_1_(*x*), *g*_2_(*x*),…, *g*_*n*_(*x*) are *n* objective functions. The objective function problem is solved using BFOA algorithm.

(7) $$\begin{array}{c} \eta^{i\left(k+1,u,q\right)}=\eta^{i\left(k,u,q\right)}+B\left(i\right)\phi \left(k\right),\\ P_{c}\left(\phi \right)=\left[\sum_{k=1}^{N}Le^{-\delta \sum_{l=1}^{n}{\left(\phi l+\phi l\right)}^{2}}\right]-\left[\sum_{k=1}^{N}Me^{-\delta \sum_{l=1}^{n}{\left(\phi l+\phi l\right)}^{2}}\right], \end{array}$$

where *L* represents the current fitness value of, “*M*” represents the average fitness value of the *l*  iteration, and (*k*) marks the distance value at each angle duration.

This is accomplished by using

(8) $$\begin{array}{c} Fs=G\left(\left(pc\left(\phi \right)\right)\right). \end{array}$$

Optimal features are selected by using the best fitness value obtained using the MBFOA algorithm [30]. (Algorithm 1)

**Figure 2:**
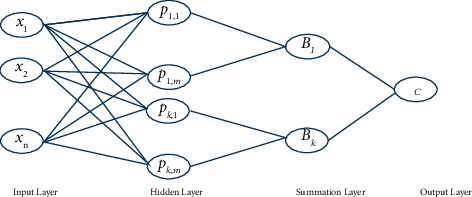
Probabilistic neural network.

The probabilistic neural network has a multilayer design that feeds forward data as shown in Figure 2. Implementing the applied arithmetic rule known as kernel discriminate analysis is what is meant when we talk about PNN's definition [31]. The processes in this are structured into a multilayered feedforward network, which consists of four layers: the input layer, the pattern layer, the summation layer, and the output layer [32]. PNN categories have several benefits, the most important of which are rapid training methods, a structure that is inherently parallel, and a guarantee that they will converge to the best possible classifier as the size of the representative training set continues to grow. Additionally, training samples can typically be added or removed without requiring extensive retraining [33]. A PNN is consequently superior to numerous models of neural networks in terms of its learning speed, and it is effective in a wide range of applications [34].

### 3.3. Retrieval

A comparable segment of the audio file must be retrieved in order to complete the audio retrieval process. The process of extracting features is the most important step in the audio recovery operation. The raw audio file may be difficult to work with, which is why this function displays the numerical representation of an audio file instead [35]. The features are pulled out of each audio file in the database and saved in the feature database once they have been extracted. The query audio file has its features extracted, and then those characteristics have to be compared with the features of every audio file that is stored in the feature database. If a query audio feature is found to be compatible with the feature database, the audio file that corresponds to the query may be obtained.

The distance between the query example and the individual samples is immediately used by the easy retrieval strategy. Additionally, the retrieval list that supports the measured distance is provided here for your convenience [36]. It is checked against the whole database, many relevant audios are obtained, and the retrieval pace is sluggish. This is especially true when the growing database is quite vast. It is recommended to use a PNN classification in conjunction with a Euclidean distance measure as a retrieval strategy in order to increase the speed of the search and return a variety of files that are linked to the query [37].

Using the probabilities derived from PNN, the sound in question is assigned to one of the two primary categories—namely speech or music—using the hierarchical retrieval process described above. As the pattern layer is being produced, a calculation is made to determine the probability density function for a single [38]. This is accomplished using:

(9) $$\begin{array}{c} F\left(Y\right)=\frac{1}{{\left(2\pi \right)}^{d/2\cdot \sigma^{d}}}e^{-{\left\|Y-Y_{j}\right\|}^{2}/2\sigma^{2}},\\ G_{i}\left(Y\right)=\frac{1}{{\left(2\pi \right)}^{d/2\cdot \sigma^{d}}}\frac{1}{N_{i}}\sum_{j=1}^{N_{i}}e^{-{\left\|Y-Y_{j}\right\|}^{2}/2\sigma^{2}},\\ g_{i}=\overset{P}{\underset{p=1}{\sum }}\overset{M}{\underset{m=1}{\sum }}\left(\frac{\partial x_{p,m}}{\partial t_{i}}e_{p,m}\right). \end{array}$$

**Figure 3:**
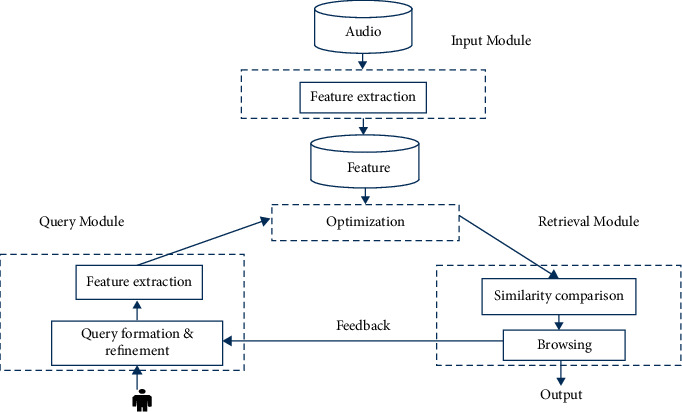
Proposed algorithm stages.

Following that, the distances between the query and the samples were measured using the category rather than the full database, and an ascending distance list was created as the outcome of the retrieval process. With the help of this method, it is possible to stop the processing of a number of irrelevant files sooner after the search has been started [39]. To get the files that are relevant to the query file, the Euclidean distance computation and relevance matching are both carried out. The steps involved in the recovery of the audio are shown in Figure 3. Equation (9) is the formula that may be used to determine the Euclidean distance, and its definition is as follows: The distance between the two places calculated using the Euclidean method.

(10) $$\begin{array}{c} P=\left(p_{1},p_{2}\ldots p_{n}\right)\text{ and }Q=\left(q_{1},q_{2}\ldots q_{n}\right),\;\sqrt{{\left(p_{1}-q_{1}\right)}^{2}+{\left(p_{2}-q_{2}\right)}^{2}+\cdots +{\left(p_{n}-q_{n}\right)}^{2}}\;. \end{array}$$

In order to define the class label, the category of the audio signal must first be obtained. A comparison is made between the provided dataset and the training set of features in order to get data from the given dataset. Because the structured database is based on the findings of the audio categorization, it is possible to obtain both the audio signal that was recovered and the category to which the audio belongs. The audio signal that is included inside the class will be displayed to the user once it has been ranked according to how closely it matches the query signal [40]. In the first step of the testing process, it is determined if the provided testing feature is music or voice. If it is determined that the signal is in fact related to music, the label will be categorized as one of the following instruments: cello, clarinet, flute, guitar, organ, piano, saxophone, trumpet, violin, and band. If the testing characteristic is found to be a speech, the voice will be classified as either male or female once again [41].

## 4. Result and Discussion

The suggested method is evaluated with the use of the audio dataset, which contains a collection of music, voice, and instrumental signals compiled by marsyasweb [42] and ffuhrmann [43]. The dataset contains a total of 128 sounds, 64 of which are musical and 64 of which are vocal. Audio files and 1100 instrumental signal audio files are organized into 11 categories, including cello (100), clarinet (100), flute (100), acoustic guitar (100), electric guitar (100), organ (100), piano (100), saxophone (100), trumpet (100), violin (100), and singing voice audio files (100). A training set consisting of eighty percent of the audio recordings in the dataset is chosen, and the remaining twenty percent of the audio files are assigned to the test set. The task that was suggested was carried out in the context of MATLAB [44]. The signal characteristics and audio signal that were used in the work being suggested are shown in Tables 1–3.

**Figure 4:**
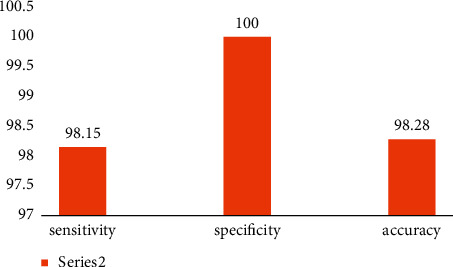
Sensitivity, specificity, and accuracy analysis.

Figure 4 shows the comparative sensitivity, specificity, and accuracy of the prediction rate.

(11) $$\begin{array}{c} \text{Sensitivity}=\frac{TP}{\left(TP+FN\right)}\\ =\frac{\left(\text{Number of true positive}\right)}{\left(\text{Number of all positive}\right)},\\ \text{Specificity}=\frac{TN}{\left(TN+FP\right)}\\ =\frac{\left(\text{Number of true negative}\right)}{\left(\text{Number of all negative}\right)},\\ \text{Accuracy}=\frac{\left(TN+TP\right)}{\left(TN+TP+FN+FP\right)}\\ =\frac{\left(\text{Number of correct}\right)}{\left(\text{Number of all}\right)}. \end{array}$$

It is clear from looking at this figure that the audio classification and retrieval approach based on MBFOA is capable of achieving high levels of sensitivity, specificity, and accuracy. After doing a side-by-side comparison of these benchmark performance results with our own, our team was able to determine that this was audio [45].

The proportion of impostors whose biometric data are accepted by the system is referred to as the false acceptance rate (FAR). In this identification system based on biometrics, users are not required to make any assertions about their identities. As a result, this percentage has to be as low as it can possibly be in order for the system to be unable to accept the individual who is not already registered in the system. Therefore, there should be as few false acceptances as possible in comparison to false rejections [46].

False rejection rate (FRR) is the proportion of legitimate users who are unable to log in as a result of the biometric technology that is being used. Because the user will be making assertions about their identification inside the biometric verification system, the system must ensure that it does not reject an enrolled user, and the frequency of false rejections must be reduced to the greatest extent feasible [17]. Therefore, the number of false rejections should be kept to a minimum in comparison to the number of false acceptances.

Genuine acceptance rate (GAR) is the proportion of genuine users that are accepted by the system. GAR = 100 − FRR is supplied.

**Figure 5:**
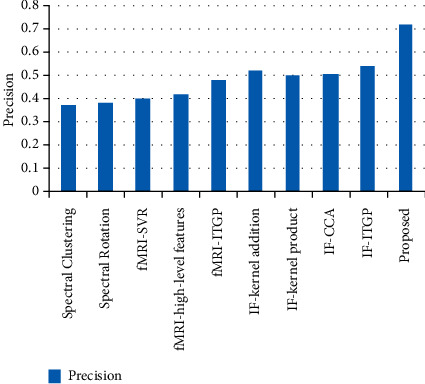
Average precision.

**Figure 6:**
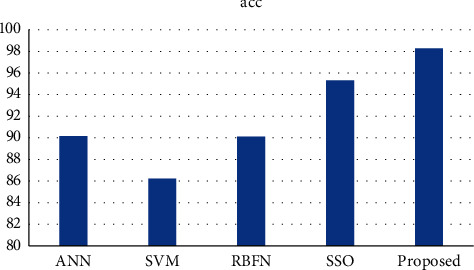
Comparative analysis proposed approach (accuracy rate with existing approach rates).

It has been determined that there has been a reduction in the GAR, FAR, FRR, and error rate. Based on these findings, it is clear that the characteristics that do not work well for classification have been successfully eliminated from the feature selection model.  Table 4 displays the results of the categorization that was performed.  Table 5 contains an examination of the precision as well as the recall that was performed.  Figure 5 depicts a comparison of the planned work's average precision with the acoustic characteristics that already exist. [47] The signal classification approach that may be used for multimedia applications performs much better than the benchmark findings. As a consequence, when categorizing audio signals, we may infer that this result is superior to the standard that is currently being used in the industry.  Figure 6 depicts the percentage of accuracy achieved by using the currently available methods [27].


(12) $$\begin{array}{c} \text{ Precision }=\;\frac{\text{ Numberofrelevantaudio }}{\text{Numberofretrievedaudio}}\text{ and Recall }=\;\frac{\text{Numberofrelevantaudio}}{\text{ Totalmumberofretrievedaudio }}\;. \end{array}$$

The confusion matrix for the musical data is shown in Table 5. In the confusion matrix, each row represents the examples that fall into the category of an actual class, while each column represents the occurrences that fall into the category of a predicted class [30]. The feature vector for this experiment work was derived from the initial feature set, which consisted of a total of 41 features.

**Figure 7:**
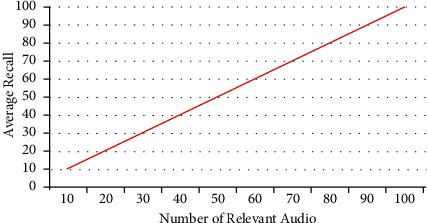
Average recall rate.

The average recall rate has been calculated for all the given query audio files and the results are shown in Figure 7, indicating that the recall rate will be higher after a certain number of relevant audio files.

## 5. Conclusions

An efficient audio data classification and retrieval approach that may partition the audio stream into similar pieces is explained in the proposed work. These areas are separated into music and speech, and if the signal is a voice, it is further categorized as either a male or female voice. In the event if the signal is music, it is broken down into the following categories: cello, clarinet, flute, guitar, organ, piano, saxophone, trumpet, violin, and the band. When it came to categorizing and retrieving audio recordings, a strategy that was based on MBFOA was used. To begin, the noise that was present in the signal that was collected is removed by applying the mean filter at the point where it filters the audio signal. It is much simpler to exclude the silence or other unimportant frequency characteristics from the audio signal input by using a procedure that utilizes peak estimation and pitch extraction. Thereafter, the MBFOA is used to pick the ideal testing feature, and the PNN classifier input chosen feature is used to classify the data based on that feature. During the retrieval step, the characteristics that correspond to the category that was acquired as a consequence of the classification process will be retrieved and listed. In the not too distant future, the audio signal will be differentiated from the continuous audio stream. Our method for categorizing sounds will be improved in the near future so that it can categorize sounds into a greater number of sound categories [48].

## Figures and Tables

**Algorithm 1:**
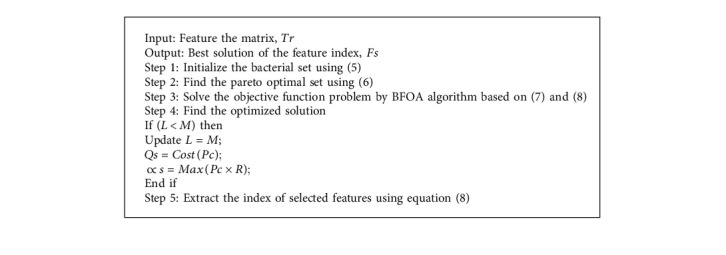
MBFOA algorithm.

**Table 1:** A summary of the properties of audio signals.

Audio property	Value
Audio bit rate	352kbps
Audio format	.wav
Audio length	30sec
Audio sample size	16 bit
Channel	Mono

**Table 2:** Audio database structure (marsyasweb).

Sl. No.	Class name		No. of files
1	Music		64
2	Speech	Male	41
3		Female	23
Total			128

**Table 3:** Performance metrics calculation.

Class	Cel	Cla	Flu	Gac	Gel	Org	Pia	Sax	Tru	Vio	Voi
Precision	98	99	98	98	98	98	98	99	98	98	99
Recall	98	99	98	98	98	98	98	99	98	98	99

**Table 4:** Average values for GAR, FAR, FRR, accuracy, and error.

Overall accuracy (%)	
Parameter	With optimization
Average GAR	100
Average FAR	0
Average FRR	0
Average accuracy	98.28
Average error rate	1.72

**Table 5:** Confusion matrix of the proposed method.

Actual predicted	Cel	Cla	Flu	Gac	Gel	Org	Pia	Sax	Tru	Vio	Voi
Cel	98	0	1	0	0	1	0	0	0	0	0
Cla	0	99	0	1	0	0	0	0	0	0	0
Flu	1	0	98	0	0	0	0	0	1	0	0
Gac	0	1	0	98	0	0	0	0	0	0	1
Gel	1	0	0	0	98	0	1	0	0	0	0
Org	0	0	0	0	1	98	0	0	0	1	0
Pia	0	0	0	0	0	0	98	1	0	1	0
Sax	0	0	0	0	1	0	0	99	0	0	0
Tru	0	0	1	0	0	0	1	0	98	0	0
Vio	0	0	0	0	0	1	0	0	1	98	0
Voi	0	0	0	1	0	0	0	0	0	0	99

## Data Availability

The data that support the findings of this study are available from the corresponding author upon request.
